# Electronic Health Literacy and Dietary Behaviors in Taiwanese College Students: Cross-Sectional Study

**DOI:** 10.2196/13140

**Published:** 2019-11-26

**Authors:** Shu Ching Yang, Yi Fang Luo, Chia-Hsun Chiang

**Affiliations:** 1 Institute of Education National Sun Yat-Sen University Kaohsiung Province of China Taiwan

**Keywords:** college, dietary, health literacy, students

## Abstract

**Background:**

Given the recognized importance of preventing poor dietary behaviors during adolescence, we need a better understanding of college students’ dietary behaviors. Studies have found that individual factors and electronic health (eHealth) literacy may affect one’s dietary behaviors. However, few studies have fully investigated the effect of the three levels of eHealth literacy (functional, interactive, and critical) and the interactive effect of individual factors (eg, gender, monthly expenses, and frequency of cooking) and the three levels of eHealth literacy on the four aspects of dietary behaviors (consumer health, balanced diet, regular eating habits, and unhealthy food intake).

**Objective:**

This study aimed to investigate whether individual differences and higher eHealth literacy are associated with more positive dietary behaviors and less unhealthy dietary intake.

**Methods:**

The eHealth Literacy Scale is a 12-item instrument designed to measure college students’ functional, interactive, and critical eHealth literacy. The Dietary Behaviors Scale is a 14-item instrument developed to measure four aspects of dietary behaviors of college students. A questionnaire was administered to collect background information about participants’ gender, monthly expenses, and frequency of cooking. A national sample of college students was surveyed, and 813 responses were obtained. We conducted a multiple regression analysis to examine the association among individual factors, eHealth literacy, and dietary behaviors.

**Results:**

This study found that functional eHealth literacy was negatively related to unhealthy food intake (beta=−.11; *P*=.01), and interactive eHealth literacy was positively related to balanced diet (beta=.25; *P*<.001) and consumer health (beta=.15; *P*=.02). Moreover, critical eHealth literacy was positively related to consumer health (beta=.30; *P*<.001) and regular eating habits (beta=.20; *P*=.002). Finally, the interactive effect between gender and interactive eHealth literacy was negatively related to balanced diet (beta=−.22; *P*<.001). The interactive effect between monthly expenses and functional eHealth literacy was positively related to balanced diet (beta=.07; *P*=.03), although the interactive effect between monthly expenses and critical eHealth literacy was negatively related to balanced diet (beta=−.10; *P*=.047).

**Conclusions:**

This study showed that Taiwanese college students with higher functional eHealth literacy were more likely to engage in fewer unhealthy food consumption practices. Those who had higher interactive and critical eHealth literacy were more likely to engage in positive dietary behaviors than those with functional eHealth literacy. Surprisingly, females with high interactive eHealth literacy were more likely to have a poor balanced diet. In contrast, students with higher monthly expenses and higher functional eHealth literacy were more likely to have a balanced diet. However, students with higher monthly expenses and higher critical eHealth literacy were less likely to maintain a balanced diet.

## Introduction

### Background

The importance of preventing poor dietary behaviors during adolescence has been recognized because of its impact on long-term health issues such as the development of obesity and other noncommunicable diseases [1]. Students’ dietary behaviors are most affected by their families and schools before they start college, but afterward, their dietary choices are mostly made independently [2]. The college years offer an opportunity for new experiences and personal freedom, such as the autonomy of food choices; however, the period is also noted for the emergence of unhealthy behaviors that place college students at risk of health problems [3]. Noteworthy findings about college students’ unhealthy eating behaviors reveal frequent consumption of snacks and fried foods and of fewer than 5 servings of fruits and vegetables daily [4,5]. Moreover, college students may put themselves at increased risk by consuming unsafe foods that may cause foodborne illness (eg, undercooked meat and shellfish containing marine toxins) or by not following accepted food safety practices [6]. Clearly, campus life should provide college students with crucial opportunities to develop healthy eating behaviors and adopt a nutritious and balanced food intake, thereby forming a strong basis for good health throughout life. Thus, we need a better understanding of college students’ dietary behaviors.

Dietary behaviors, including consumer health, balanced diet, regular eating habits, and unhealthy food intake, can be viewed as positive or negative actions in relation to maintaining or enhancing health [7]. Consumer health refers to individuals’ health awareness and decisions about food purchasing. A balanced diet indicates eating the recommended amount of a variety of foods from each food group daily. Regular eating habits refer to individuals’ healthy eating habits. Unhealthy food intake refers to individuals’ consumption of junk food and drinks [7]. Individuals must understand the prevailing nutritional recommendations with respect to the food products that they are considering and use such recommendations to choose suitable foods [8]. These practices and skills are included in the concept of health literacy.

Health literacy refers to an individual’s ability to access, understand, and use information to promote and maintain good health [9]. Studies have shown that individuals with higher health literacy are more likely to use food labels and have higher dietary quality [10], healthier eating behavior, and lower consumption of sugar-sweetened beverages [11]. Recently, the advent of the internet has drastically changed how health information is disseminated, and the internet is now widely used to obtain this information. People should not only be health literate but also have the capabilities, resources, and motivation to find, understand, and appraise health information using digital services and technology [12]. Unlike other distinct forms of literacy, electronic health (eHealth) literacy combines basic literacy as well as information, health, media, computer, and scientific literacies and applies them to eHealth promotion [13]. To obtain a complete overview of people’s skills in obtaining and using health information, it is more necessary to measure eHealth literacy than to measure health literacy [14].

Norman and Skinner [13] have defined eHealth literacy, which includes functional, interactive, and critical levels [15], as “the ability to seek, find, understand, and appraise health information from electronic sources and to apply the knowledge gained to addressing or solving health problems.”. Functional eHealth literacy refers to basic skills in reading and writing about Web-based health information. Interactive eHealth literacy specifies the communicative and social skills that can be used to extract information in social Web-based multimedia environments. Critical eHealth literacy involves the cognitive skills that can be applied to critically evaluate the credibility, relevance, and risks of sharing and receiving Web-based health information [16-18]. Such a classification indicates that the different levels of literacy progressively allow for greater autonomy and personal empowerment in decision making as well as engagement in a wider range of health actions [9,15]. Having the composite skills of eHealth literacy allows individuals to achieve positive health behaviors [19].

### Objectives

The integrative model of eHealth use (IMeHU) indicates that people with high eHealth literacy are not only more inclined to use the internet to find answers to health-related questions but are also able to understand the information that they find, verify its veracity, and use it to promote health behaviors [20]. Studies have found that 27 % web user reported that their eating habits had affected by eHealth resources (eg, health websites) [21]. In addition, individuals with higher eHealth literacy adopt more balanced nutrition [22]. Recently, researchers have focused on the three levels of eHealth literacy and found that individuals with high functional [16] and critical eHealth literacy [16,17] are more likely to practice balanced eating habits. However, other studies have found no statistically significant evidence of a relationship between interactive eHealth literacy and eating habits [16,17]. A systematic review demonstrated few studies reporting an association between eHealth literacy and health behaviors, with inconsistent results [19]. Accordingly, this study attempted to fully investigate the roles of functional, interactive, and critical eHealth literacy on the four aspects of dietary behaviors. We thus propose the following hypothesis: H1: college students who possess higher functional, interactive, and critical eHealth literacy will engage in more positive dietary behaviors.

The IMeHU suggests that individual factors may affect one’s health behaviors [20]. Studies have found that college students with higher meal expenses were more likely to display poor dietary behaviors [23,24]. Cooking skills are important for health and well-being [25], as studies have shown that an intervention involving gardening, nutrition, and cooking can improve dietary intake [26]. Some studies have also reflected gender differences, such as 1 study that showed girls’ higher engagement in dietary behaviors than boys [27]. However, other studies have revealed female college students’ insufficient vegetable intake [28] and a poor balanced diet and unhealthier food intake than that of male students [7]. Given the abovementioned studies on individual differences in dietary behaviors, this study performed analyses of individual factors (eg, gender, monthly expenses, and frequency of cooking) and the three levels of eHealth literacy to examine the explanatory power of the four aspects of dietary behaviors.

Similarly, little attention has been paid to the interactive effect of individual factors and the three levels of eHealth literacy on the four aspects of dietary behaviors. It is necessary to clarify the effects of individual factors and the three levels of eHealth literacy, as well as how the interactive effect of these 2 elements affects the four aspects of dietary behaviors, to develop an effective health education program to promote healthy dietary behaviors among college students. Therefore, the objective of this study was to investigate the associations among individual factors, eHealth literacy, and dietary behaviors in Taiwanese college students with the goal of determining whether high eHealth literacy is associated with more positive dietary behaviors and less unhealthy dietary intake and to investigate the interactive effects of individual factors and eHealth literacy on the four aspects of dietary behaviors. In accordance with the IMeHU [20] and the abovementioned studies, we therefore propose the following hypothesis: H2: individual factors and eHealth literacy have an interactive effect on the dietary behaviors of college students.

## Methods

### Participants

This study was conducted nationwide in Taiwan. The list of colleges came from Taiwan’s Ministry of Education. Sampling units were drawn from within each region. We contacted teachers at selected colleges to request their assistance in the pen-and-paper survey distribution. Ultimately, 1100 college students from 10 schools were recruited to participate in the survey. Of the 895 surveys received, we excluded 25 blank surveys, 34 surveys with incomplete respondents’ individual factors, and 23 surveys with fixed mode answers. However, 21 surveys with fewer than 3 items incomplete were retained. We applied expectation-maximization method of multiple imputation to the missing data. In other words, 813 surveys were statistically analyzed in this study (813/895, 90.8%). We gathered the respondents’ individual factors, including information about age, gender (male and female groups), monthly expenses (<NT $5000, NT $5000-NT $10,000, and >NT $10,001), and frequency of cooking (5-point Likert scale). The frequency of cooking was measured by asking how often the students cooked by themselves and was rated based on the responses on a scale from 1 (never) to 5 (always).

### The Survey Instrument

#### Electronic Health Literacy Scale

Participants’ eHealth literacy was evaluated using the eHealth Literacy Scale (eHLS) [17], which consists of 12 items that constitute 3 levels: functional (3 items), interactive (4 items), and critical (5 items) eHealth literacy. The functional level evaluates individuals’ basic reading and writing skills and basic knowledge of health conditions and health systems. The interactive level evaluates individuals’ communicative and social skills, which can be used to abstract information and derive meaning from different forms of communication. The critical level assesses individuals’ most advanced cognitive skills, which can be applied to critically analyze information, discern the quality of health websites, and use good information to make informed decisions about health.

The items were answered using a 5-point Likert scale, with scores ranging from 1 (total disagreement) to 5 (total agreement). High scores on the respective levels indicated higher functional, interactive, and critical eHealth literacy. Cronbach alpha is a statistic commonly quoted by authors to demonstrate that tests and scales that have been constructed or adopted for research projects are fit for the research purpose. The alpha values of .45 to .98 for this instrument have been described as acceptable [29]. Within the sample used in this study, the Cronbach alpha values of functional eHealth literacy, interactive eHealth literacy, and critical eHealth literacy were .81, .87, and .90, respectively, indicating that the eHLS had acceptable internal reliability.

#### Dietary Behaviors Scale

The participants’ dietary behaviors were evaluated using the Dietary Behaviors Scale (DBS) [7]. Using item analyses, exploratory factor analysis, and confirmatory factor analysis, Luo et al [7] revealed that the DBS is a reliable and validated measure of dietary behaviors of Taiwanese college students. The DBS consists of 14 items that comprise 4 aspects: consumer health (4 items), balanced diet (4 items), regular eating habits (3 items), and unhealthy food intake (3 items). The consumer health aspect evaluates individuals’ attitudes and decisions about food purchasing. The balanced diet aspect evaluates individuals’ nutrient intake. The regular eating habits aspect evaluates individuals’ healthy eating habits. The unhealthy food intake aspect evaluates individuals’ consumption of food and drinks that are high in calories, fat, salt, or sugar.

The items were answered using a 5-point Likert scale, with scores ranging from 1 (never) to 5 (always). High scores in the individual aspects indicated positive attitudes and decisions about product purchases, more balanced and regular eating habits, and more consumption of unhealthy food. Within the sample used in this study, the Cronbach alpha values of consumer health, balanced diet, regular eating habits, and unhealthy food intake were .82, .72, .59, and .68, respectively, indicating that the DBS had acceptable internal reliability [29].

### Data Analysis

Analyses were conducted using SPSS 17.0 (IBM Corp). We performed 4 multiple regression analyses to examine the explanatory power of the four aspects of dietary behaviors. In the model, individual factors, the 3 levels of eHealth literacy, and the interaction of these 2 elements were entered. Gender was viewed as a dummy variable where males were coded as 0, and monthly expenses and frequency of cooking were viewed as continuous variables.

### Ethical Considerations

The study adopted an anonymous questionnaire in line with the government’s institutional review board rules for exempt review. The questionnaire instructions informed the participants of the research purpose and confidentiality and indicated that they had the right to refuse to participate at any time. The participants received the questionnaire and gifts at the same time; even if a participant decided to drop out of the investigation, he or she still received the gifts (a pen and an L-folder). This approach was intended to be fair to each participant, to avoid the impact of gift incentives on the participants, and to provide compensation for the participants.

## Results

### Participants’ Demographics and Characteristics

Table 1 presents the demographics and characteristics of the study participants. The mean age of participants was 20.08 (SD 1.43) years. Of the 813 participants, 47.1% (383/813) were female and 40.7% (331/813) reported that their monthly expenses were less than NT $5000. The mean frequency of cooking of the participants was 2.23 (SD 0.96).

**Table 1:** Demographics and characteristics of the sample.

Variables		n (%)
**Gender**		
	Male	430 (52.9)
	Female	383 (47.1)
**Monthly expenses**		
	<NT $5000	331 (40.7)
	NT $5001-NT $10,000	409 (50.3)
	>NT $10,001	73 (9.0)
**Frequency of cooking**		
	Never	180 (22.2)
	Seldom	373 (45.9)
	Sometimes	166 (20.4)
	Often	80 (9.8)
	Always	14 (1.7)

### Descriptive Statistics of Electronic Health Literacy and Dietary Behaviors

Among all participants, the mean scores of functional eHealth literacy, interactive eHealth literacy, and critical eHealth literacy were 3.56 (SD 0.77), 3.57 (SD 0.71), and 3.59 (SD 0.72), respectively, indicating that college students had medium or higher levels of eHealth literacy.

On the DBS, the mean scores of the regular eating habits, balanced diet, unhealthy food intake, and consumer health were 3.15 (SD 0.73), 2.92 (SD 0.70), 3.02 (SD 0.72), and 3.12 (SD 0.76), respectively. This result indicated that college students had positive consumer health and regular eating habits although they did not maintain a balanced diet. Moreover, they sometimes ate unhealthy food.

### Analysis of Electronic Health Literacy and Dietary Behaviors

In the multiple regression analysis, first, the tolerance and variance inflation factor (VIF), as 2 collinearity diagnostic factors, were examined to assess multicollinearity in this study. The collinearity diagnostics results showed that tolerance ranged from 0.28 to 0.99, and VIF ranged from 1.01 to 3.78. These results indicate that explanatory variables in this multiple regression model were weakly linearly related [30].

The results of the multiple regression analysis are displayed in Multimedia Appendix 1, which shows that functional eHealth literacy was negatively related to unhealthy food intake (beta=−.11; *P*=.01), and interactive eHealth literacy was positively related to balanced diet (beta=.25; *P*<.001) and consumer health (beta=.15; *P*=.02). Moreover, critical eHealth literacy was positively related to consumer health (beta=.30; *P*<.001) and regular eating habits (beta=.20; *P*=.002). Thus, hypothesis 1 was partially supported.

The study found that the interactive effect between gender and interactive eHealth literacy was negatively related to balanced diet (beta=−.22; *P*<.001). The interactive effect between monthly expenses and functional eHealth literacy was positively related to balanced diet (beta=.07; *P*=.03), although the interactive effect between monthly expenses and critical eHealth literacy was negatively related to balanced diet (beta=−.10; *P*=.047). Therefore, hypothesis 2 was partially supported.

## Discussion

### Principal Findings

This study attempted to fully investigate the associations among a broader concept of eHealth literacy and dietary behaviors and to examine the interactive effects of individual factors and the three levels of eHealth literacy on the four aspects of dietary behaviors. We used the IMeHU to explore the associations among individual factors, eHealth literacy, and dietary behaviors. The study found a statistically significant association among the three levels of eHealth literacy and the four aspects of dietary behaviors. Furthermore, there was an interactive effect between individual factors and eHealth literacy on the balanced diet aspect of dietary behaviors.

This study demonstrated that functional eHealth literacy was negatively related to unhealthy food intake; however, interactive and critical eHealth literacy were not related to unhealthy food intake. This result indicates that the impact of functional eHealth literacy is greater than that of interactive critical eHealth literacy on unhealthy food intake among Taiwanese college students. Previous studies have found that individuals with adequate functional health literacy are less likely to consume fried chicken [31] and sugar-sweetened beverages [11]. Moreover, studies have indicated that targeted intervention strategies that address health literacy, such as quantitative health information guides, are advantageous to reduce sugar-sweetened beverage consumption [32]. Functional eHealth literacy involves basic skills in reading and writing about Web-based health information [17,18]; thus, enabling Taiwanese college students to understand the risks of unhealthy food intake and engage in fewer unhealthy food consumption practices is important.

Consistent with previous studies [19,20], this study showed that interactive eHealth literacy was positively related to the balanced diet and consumer health aspects of dietary behaviors, and critical eHealth literacy was positively related to regular eating habits and consumer health. To our knowledge, this study was the first to investigate the association among the three levels of eHealth literacy and consumer health behaviors. The Taiwanese government has enacted a food education curriculum to improve knowledge of and skills in healthy consumption and to empower consumers to make healthy choices about food and diet. Interactive eHealth literacy involves more advanced cognitive and literacy skills that can be used to actively participate in everyday activities [15] and to promote healthy consumption patterns [33,34]; thus, enriching interactive eHealth literacy might help Taiwanese college students to engage in balanced diet and consumer health practices. Critical eHealth literacy involves the cognitive skills necessary to critically evaluate Web-based health information [16-18] and use such information to make informed decisions about health [16,17]. Critical eHealth literacy allows individuals to evaluate health issues and recognize risks and benefits as well as to advocate for themselves [35], thereby facilitating Taiwanese college students’ regular healthy eating habits and consumer health practices.

Perhaps surprisingly, the study found that females with high interactive eHealth literacy were more likely to have a poor balanced diet. Previous studies in Taiwan have also shown female college students’ insufficient vegetable intake [28] and a poor balanced diet [7]. It is inferred that social culture plays a central role in the lives of adolescent girls and young women and may influence their female body image and perception of beauty [28]. Although obesity prevalence is quite low among Taiwanese girls, the pressures to be thin still seem to be profound [36]. Gender differences in food choices, therefore, appear to be partly attributable to women’s greater weight control involvement [37]. In addition, interactive health communication applications have great potential to improve health, but they may also cause harm [38]. The internet hosts a wide variety of weight loss diets for which individuals may search, but the effects such diets claim may be unconfirmed or exaggerated, false, or even harmful. In addition, more than 15% of internet users have reported feeling overwhelmed and confused by the amount of information available Web-based [39]. Thus, females with high interactive eHealth literacy might be misled by false information and choose unhealthy meals or portions to lose weight, leading to their poor balanced diet. Therefore, better-designed studies are needed to confirm the dieting attitudes and behaviors of females with high interactive eHealth literacy, how eHealth literacy and their body image shape their perceptions, and how body satisfaction may mediate the association between dieting behaviors and health attitudes through their use of Web-based health information.

Consistent with previous studies [23,24], this study found that Taiwanese college students with high monthly expenses were more likely to demonstrate irregular eating habits and consumption of unhealthy food. When living in a student residence, college students become more self-dependent, which also implies that price and budget become increasingly important [40]. College students with a limited budget must choose their food cautiously [24], which can enable regular eating habits and less consumption of snacks and sugary drinks. In contrast, college students with a higher budget might be able to engage in more hedonic eating, resulting in a poor dietary intake that might have a harmful impact on their health and well-being. Paradoxically, this study found that students with higher monthly expenses and higher functional eHealth literacy were more likely to consume a balanced diet, whereas students with higher monthly expenses and higher critical eHealth literacy were less likely to maintain a balanced diet. Therefore, multilevel nutritional interventions may be beneficial to promote healthy eating behavior and dietary intake, particularly among students with high monthly expenses. Schools should provide more nutrition information about food to enhance students’ functional eHealth literacy. Future qualitative study is also needed to further examine a broad range of factors that might influence how the level of nutrition-related knowledge and perceptions impact the development of eating patterns among students with high monthly expenses.

### Limitations

This study is not without limitations. First, we did not remove responses with little missing or incomplete data because those with very little missing data can still be part of the analysis. Second, unhealthy food intake was measured based on frequency (from seldom to always) rather than an average of kilocalories and consumption per day. Thus, our measure of unhealthy food intake may not accurately indicate the effect of interactive and critical eHealth literacy on unhealthy food intake. Third, the study sample was educated and age restricted in Taiwan. Thus, the findings should not be overgeneralized and should be interpreted in light of the sample’s homogeneity. Finally, because the study presented some interesting and paradoxical findings, along with the associations among these factors, eHealth literacy, and dietary behaviors among college students, further studies should examine a broad range of intrapersonal (eg, taste preference, family eating habits, and level of nutrition-related knowledge), interpersonal (eg, peer influence and campus lifestyle), cultural (eg, media and academic activity), and environmental factors (food availability and food cost) as well as social norms and beliefs that might be influential in determining college students’ eating behavior and dietary intake.

### Conclusions

This study, to our knowledge, was the first to establish an association among individual factors, the 3 levels of eHealth literacy, and the four aspects of dietary behaviors among college students. This study found that Taiwanese college students with interactive and critical eHealth literacy were more likely to engage in positive dietary behaviors than those with functional eHealth literacy. For the group of students with high monthly expenses, the role of functional eHealth literacy was greater than that of critical eHealth literacy in balanced diet. Moreover, Taiwanese college students with functional eHealth literacy were less likely to engage in unhealthy food intake. These findings have important implications for health educators, who should provide college students with information to help them understand the risks of high-calorie, high-fat, high-salt, or high-sugar diets and thereby reduce their consumption of unhealthy food. As an interactive effect between gender and interactive eHealth literacy on balanced diet was identified, further research is needed to help female college students critically evaluate the credibility and risks of Web-based health information about losing weight and thereby participate in healthy eating practices.

## Appendix

Multimedia Appendix 1 Multiple regression analysis of the four aspects of dietary behaviors.

## Abbreviations

DBS: Dietary Behaviors Scale
eHealth: electronic health
eHLS: eHealth Literacy Scale
IMeHU: integrative model of eHealth use
VIF: variance inflation factor
