# Supplementary material for: Electronic Health Literacy and Dietary Behaviors in Taiwanese College Students: Cross-Sectional Study
Source: J Med Internet Res. 2019 Nov 26;21(11):e13140. doi: 10.2196/13140 (PMC6904901; doi:10.2196/13140)
Supplement: Multimedia Appendix 1 [file jmir_v21i11e13140_app1.docx]

| Variable | Regular eating habits | | | | | | Balanced diet | | | | | | Unhealthy food intake | | | | | | Consumer health | | | | | |
| --- | --- | --- | --- | --- | --- | --- | --- | --- | --- | --- | --- | --- | --- | --- | --- | --- | --- | --- | --- | --- | --- | --- | --- | --- |
|  | *B* | Beta | *P* value | *R* | *R*^2^ | *F* test (df) | *B* | Beta | *P* value | *R* | *R*^2^ | *F* test (df) | *B* | Beta | *P* value | *R* | *R*^2^ | *F* test (df) | *B* | Beta | *P* value | *R* | *R*^2^ | *F* test (df) |
| Model |  |  |  | 0.31 | 0.09 | 5.54 (15, 797) |  |  |  | 0.38 | 0.15 | 9.17 (15, 797) |  |  |  | 0.22 | 0.05 | 2.67 (15, 797) |  |  |  | 0.43 | 0.19 | 12.27 (15, 797) |
| Gender | 0.19^a^ | .13^a^ | <.001^a^ | —^b^ | — | — | −0.25 | −.18 | <.001^a^ | — | — | — | 0.06 | .04 | .26 | — | — | — | −0.01 | −.01 | .83 | — | — | — |
| Monthly expenses | −0.11^a^ | −.10^a^ | .004^a^ | — | — | — | −0.04 | −.04 | .28 | — | — | — | 0.10^a^ | .08^a^ | .02^a^ | — | — | — | −0.02 | −.02 | .57 | — | — | — |
| Frequency of cooking | 0.01 | .02 | .65 | — | — | — | 0.09 | .12 | <.001^a^ | — | — | — | −0.07^a^ | −.09^a^ | .01^a^ | — | — | — | 0.09^a^ | .12^a^ | <.001^a^ | — | — | — |
| Functional | −0.07 | −.07 | .11 | — | — | — | −0.04 | −.05 | .26 | — | — | — | −0.10^a^ | −.11^a^ | .01^a^ | — | — | — | −0.06 | −.06 | .12 | — | — | — |
| Interactive | 0.08 | .08 | .22 | — | — | — | 0.24^a^ | .25^a^ | <.001^a^ | — | — | — | 0.07 | .07 | .28 | — | — | — | 0.16^a^ | .15^a^ | .02^a^ | — | — | — |
| Critical | 0.21^a^ | .20^a^ | .002^a^ | — | — | — | 0.10 | .11 | .10 | — | — | — | 0.12 | .12 | .08 | — | — | — | 0.31^a^ | .30^a^ | <.001^a^ | — | — | — |
| Gender×functional | 0.08 | .05 | .24 | — | — | — | 0.08 | .06 | .18 | — | — | — | 0.01 | .01 | .83 | — | — | — | 0.04 | .03 | .52 | — | — | — |
| Gender×interactive | −0.04 | −.03 | .68 | — | — | — | −0.33^a^ | −.22^a^ | <.001^a^ | — | — | — | −0.11 | −.07 | .29 | — | — | — | −0.03 | −.02 | .76 | — | — | — |
| Gender×critical | −0.02 | −.01 | .86 | — | — | — | 0.15 | .10 | .12 | — | — | — | −0.08 | −.05 | .45 | — | — | — | 0.00 | .00 | .96 | — | — | — |
| Monthly expenses×functional | 0.08 | .05 | .12 | — | — | — | 0.10^a^ | .07^a^ | .03^a^ | — | — | — | −0.02 | −.02 | .65 | — | — | — | 0.05 | .03 | .31 | — | — | — |
| Monthly expenses×interactive | 0.06 | .04 | .46 | — | — | — | 0.14 | .10 | .06 | — | — | — | 0.05 | .03 | .54 | — | — | — | 0.04 | .02 | .62 | — | — | — |
| Monthly expenses×critical | −0.09 | −.06 | .30 | — | — | — | −0.15^a^ | −.10^a^ | .047^a^ | — | — | — | −0.07 | −.05 | .37 | — | — | — | −0.04 | −.03 | .60 | — | — | — |
| Frequency of cooking×functional | 0.03 | .03 | .37 | — | — | — | 0.02 | .02 | .58 | — | — | — | 0.02 | .02 | .58 | — | — | — | 0.02 | .02 | .62 | — | — | — |
| Frequency of cooking×interactive | −0.05 | −.05 | .29 | — | — | — | 0.02 | .02 | .61 | — | — | — | −0.01 | −.01 | .89 | — | — | — | 0.02 | .02 | .71 | — | — | — |
| Frequency of cooking×critical | 0.05 | .05 | .32 | — | — | — | 0.002 | .002 | .96 | — | — | — | −0.02 | −.02 | .70 | — | — | — | 0.002 | .002 | .97 | — | — | — |

^a^Significant values.

^b^ not applicabl
